# Emergency Physician Observations and Attitudes on Law Enforcement Activities in the Emergency Department

**DOI:** 10.5811/westjem.2022.12.57098

**Published:** 2023-02-20

**Authors:** Utsha G. Khatri, Elinore J. Kaufman, Emily F. Seeburger, Rucha Alur, Lynne D. Richardson, Eugenia C. South, Sara F. Jacoby

**Affiliations:** * Icahn School of Medicine at Mount Sinai, Department of Emergency Medicine, New York, New York; † Icahn School of Medicine at Mount Sinai, Institute for Health Equity Research, Department of Population Health Science and Policy, New York, New York; ‡ University of Pennsylvania Perelman School of Medicine, Division of Traumatology, Surgical Critical Care, and Emergency Surgery, Philadelphia, Pennsylvania; § Perelman School of Medicine at the University of Pennsylvania, Department of Emergency Medicine, Philadelphia, Pennsylvania; ¶ University of Pennsylvania School of Nursing, Department of Family and Community Health, Philadelphia, Pennsylvania; || University of Pennsylvania Perelman School of Medicine, Philadelphia, Pennsylvania; # Perelman School of Medicine at the University of Pennsylvania, Penn Urban Health Lab, Department of Emergency Medicine, Philadelphia, Pennsylvania

## Abstract

**Introduction:**

Law enforcement officers (LEO) interact with patients and clinicians in the emergency department (ED) for many reasons. There is no current consensus on what should comprise, or how to best enact, guidelines that ideally balance LEO activities in the service of public safety with patient health, autonomy, and privacy. The purpose of this study was to explore how a national sample of emergency physicians (EP) perceives activities of LEOs during the delivery of emergency medical care.

**Methods:**

Members of the Emergency Medicine Practice Research Network (EMPRN) were recruited via an email-delivered, anonymous survey that elicited experiences, perceptions, and knowledge of policies that guide interactions with LEOs in the ED. The survey included multiple-choice items, which we analyzed descriptively, and open-ended questions, which we analyzed using qualitative content analysis.

**Results:**

Of 765 EPs in the EMPRN, 141 (18.4%) completed the survey. Respondents represented diverse locations and years in practice. A total of 113 (82%) respondents were White, and 114 (81%) were male. Over a third reported LEO presence in the ED on a daily basis. A majority (62%) perceived LEO presence as helpful for clinicians and clinical practice. When asked about the factors deemed highly important in allowing LEOs to access patients during care, 75% reported patients’ potential as a threat to public safety. A small minority of respondents (12%) considered the patients’ consent or preference to interact with LEOs. While 86% of EPs felt that information-gathering by LEO was appropriate in the ED setting, only 13% were aware of policy to guide these decisions. Perceived barriers to implementation of policy in this area included: issues of enforcement; leadership; education; operational challenges; and potential negative consequences.

**Conclusion:**

Future research is warranted to explore how policies and practices that guide intersections between emergency medical care and law enforcement impact patients, clinicians, and the communities that health systems serve.

## INTRODUCTION

### Background

The emergency department (ED) holds a unique position at the intersection of health and society. It is the “safety net” infrastructure for acute healthcare systems across the United States (US) and a frequent entry point into healthcare institutions.^1^ As such, it is often a window into the health impacts of social, economic, and political challenges faced by individuals and in communities.^2^ Many injuries and illnesses treated in the ED attract responses from law enforcement officers (LEO) and the larger criminal legal system. While any individual seeking care in the ED may encounter LEOs, direct contact is most common for individuals who have health emergencies associated with violence, alcohol or drug use, and psychiatric concerns, individuals under arrest or incarceration, and individuals who are identified as undocumented immigrants.

### Importance

Law enforcement officers can play multiple roles in the ED, and these vary widely by institutional and community context. They provide security and respond to calls for service from hospital staff. They may oversee patients in law enforcement custody; provide transport to the hospital; collect evidence; take accident, incident or crime reports; document injuries; and in some cases patrol crowded ED waiting rooms to maintain order.^3^,^4^ While the activities, protocols, and priorities of LEOs are generally informed by their mission to maintain public safety, the scope, legality, and details of their encounters with patients may not be well understood by healthcare personnel who are responsible for providing care to patients or by healthcare administrators who set policies and guidelines for their institutions.^3^

Scholarship on the overlap between ED care and law enforcement activity is relatively new in medical and legal studies. In her recent article in the *Harvard Law Review*, legal scholar and law professor Ji Seon Song examines the social and legal context of how policing affects people in the ED.^5^ Song reports that courts have interpreted the ED as an extension of the public arena, generally allowing police to engage in the searching and questioning of patients with only the same constraints as would apply on a city street. Song argues that this doctrine does not account for the medical vulnerability of patients in the ED and that it exacerbates racialized policing practices due to the convergence of police and marginalized groups, namely Black and other minority patients and poor patients, in safety-net EDs.

Some law enforcement activities may in fact conflict with the clinical priorities of emergency physicians (EP), nurses, and staff who are tasked with initiating life- and limb-saving interventions. Additionally, LEOs’ goals to maintain social order and enforce laws may clash with ethical imperatives that guide the practice of medicine, such as respect for individual autonomy, expectation of privacy, and the principle of non-maleficence.^6^ These conflicts may lead to violations of patient privacy, erosion of trust, and compromised clinical care.

**Population Health Research Capsule**

What do we already know about this issue?

*Clinical and ethical priorities to guide patient care intersect and may conflict with priorities of law enforcement officers (LEO) in the emergency department (ED)*.

What was the research question?

*How do emergency physicians (EP) perceive law enforcement activities during emergency medical care?*

What was the major finding of the study?

*The majority (62%) perceived LEO presence as helpful for clinicians, 75% reported patients’ potential threat to public safety was highly important in allowing LEOs to access patients, and 86% felt that information- gathering by LEO was appropriate in the ED*.

How does this improve population health?

*The lack of consensus among EPs on LEO activity in the ED highlights the need for policies that optimally protect patients while securing public safety*.

### Goals of This Investigation

Despite these complexities, there is sparse legal or institutional policy to guide EPs and other clinicians in these areas of potential conflict, leading to ad hoc, informal negotiations and decisions. The American College of Emergency Physicians (ACEP) released a position statement on law enforcement information-gathering in the ED, affirming that the EP’s fundamental responsibility is to patients and specifies the circumstances in which EPs may provide LEOs with patient information.^7^ However, research on the frequency and perceived impact of LEO presence in the ED and interactions with patients during clinical practice is sparse. In this study, we sought to explore the perceptions and policy knowledge of a national sample of EPs relevant to the activities of LEOs during the delivery of emergency medical care.

## METHODS

### Study Design and Setting

In collaboration with leadership from ACEP, the Emergency Medicine Practice Research Network (EMPRN) is a voluntary group of 765 EPs representing a broad-spectrum emergency practice who are asked to complete up to four surveys a year. A wide variety of topics are covered in the questions posed to EMPRN participants, who closely mirror the national ACEP membership in terms of gender, age, years in practice, geographic region, and practice level. We developed a survey instrument to elicit information on their experiences with and perceptions of LEOs in the ED (provided in full in Appendix A). In March 2021, this survey and three other distinct surveys were distributed via an emailed link to an online survey platform to the full membership of 765 EPs in the EMPRN. The ACEP staff compiled response data and sent our research team a limited dataset containing responses to our survey for analysis. The institutional review board at the University of Pennsylvania approved this study, and the EMPRN research section reviewed the survey instrument.

### Analysis

We descriptively analyzed the survey data, generating frequency counts and percentages of respondents who responded to each survey item. Open-ended questions were used to elicit respondents’ views of prominent barriers and facilitators to policy development and implementation for LEO activities in the ED in their practice setting. We coded this data using content analysis. The reliability of the coding scheme was supported by using two independent coders and coding comparison, wherein any discrepancies or differences in interpretation were rectified by research team review and consensus.

## RESULTS

### Characteristics of Study Subjects

The survey was completed by 141 of 765 EPs (18.4%). Of those respondents, 113 (82%) were White and 114 (81%) were male. Respondents were diverse in age and geographic location (see Table 1 and Appendix Figure 1). This broadly reflects the demographics of current ACEP membership, 26% of whom are women, and 1% and 1.5% of whom are Black or Hispanic, respectively.

### Survey Results

**Figure 1:**
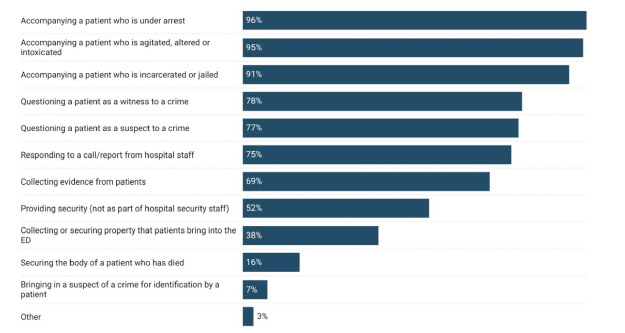
Law enforcement officer activities observed by emergency physicians in the emergency department.

When asked how frequently EPs observe LEOs interacting with ED patients, more than one third (34%) responded daily, 26% responded several times a week, and 21% responded weekly. Regarding the observed activities of LEOs in the EDs, respondents most commonly reported they observed LEOs accompanying a patient under arrest; accompanying a patient who was agitated, altered or intoxicated; or accompanying a patient who was incarcerated or jailed. More than three-fourths reported they had observed patients being questioned in the ED as a witness to a crime (78%) or a suspect in a crime (77%). (See Figure 1.)

Survey respondents described the presence of LEOs as usually or almost always helpful to their clinical work 62% of the time, while less than 2% perceived LEO presence as usually or almost always harmful to their clinical work. More specifically, respondents viewed LEO presence during clinical care as being helpful or very helpful for patients 38% of the time, for clinicians 59% of the time, and for public and community safety 65% of the time. On the other hand, respondents described LEO presence during the care of ED patients as somewhat harmful or harmful for patients 10% of the time, for clinicians 2% of the time, and for public and community safety 2% of the time.

**Figure 2:**
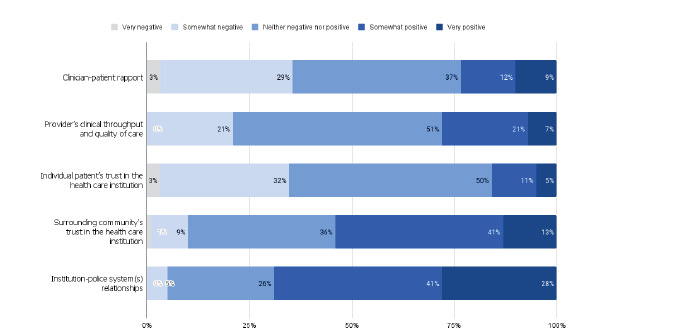
Emergency physician perceptions of the impact of law enforcement officer activity on clinical and community relationships.

There was little consensus among respondents in perceptions of how LEO presence affects multiple considerations in emergency care provision. For example, while 21% of respondents reported that LEOs very positively or somewhat positively affect clinician-patient rapport, 32% of respondents reported that the effect was somewhat or very negative. Similarly, on the topic of clinical throughput and quality of care, 28% reported a somewhat or very positive effect while 21% reported a somewhat negative effect. There was agreement on the effect of LEO presence on the surrounding community’s trust in the healthcare institution and the healthcare institution-police system relationship, as the majority of respondents reported positive impacts on both (See Figure 2).

**Figure 3:**
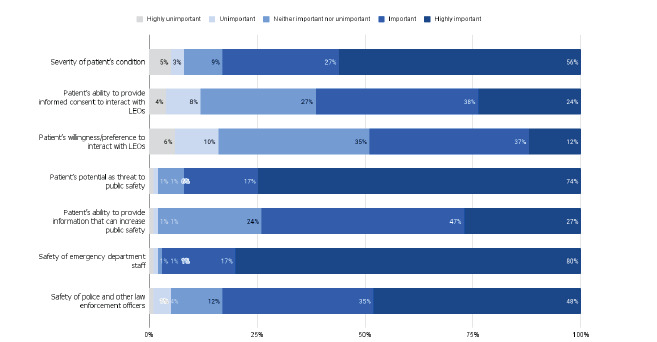
Factors influencing the emergency physician’s decision to allow law enforcement access to emergency department patients. *LEO*, law enforcement officer.

When EPs were asked about the factors highly important to determining whether to allow or not allow LEOs access to their patients, 56% of EPs reported the severity of the patient’s condition, 75% reported the patient’s potential as a threat to public safety, and 80% reported the safety of ED staff as being highly important. On the other hand, 24% thought that a patient’s ability to provide informed consent to interact with LEOs was highly important, and only 12% considered the patient’s willingness or preference to interact with LEOs as highly important (See Figure 3).

Regarding appropriateness of information-gathering about a crime or suspected crime (when safety of staff or patients is not explicitly a concern), 86% of EPs felt that it was appropriate to do so in the ED after initial work up. Only 3% reported that LEOs should not interact with patients in patient-care areas of the hospital. When asked whether they felt they had oversight or influence over LEO access to patients in the ED, EPs responded affirmatively only 54% of the time. Only 13% of EPs responded that they were aware of a policy or guideline to inform LEO interactions with ED patients. Nearly half (48%) of respondents reported they did not foresee any barriers to routine adherence, were a policy to be enacted in their ED.

### Content Analysis Results

Content analysis identified barriers and facilitators to the development and implementation of institutional policies to guide LEO activity in the ED. Five categories of barriers to policy development and adoption were identified: 1) public safety; 2) enforcement concerns; 3) difficulties related to standardization; 4) education and communication of policy; and 5) need for leadership buy-in (described in Table 2). *Public safety* referred to expressed reluctance to enforce an institutional policy that would impede the activities of LEOs. Participants raised the concern that interfering with law enforcement work could interfere with the promotion of public safety interests. *Enforcement concerns* reflected participants’ concerns that even if a policy were to exist to guide the activities of LEOs in the ED, enforcement would be challenging. Many respondents raised concerns over who in the ED would be left with the burden of enforcing the policy, and some predicted that LEOs would ignore the policy even if one existed. *Difficulties related to standardization* recognized that the nuanced nature and diverse drivers of LEO activity in the ED would be difficult to capture in a single policy. In this category, respondents raised concerns that drafting an overarching policy would be difficult due to the unique situations that arise and the time-sensitive nature of LEO activities. *Education and communication of policy* reflected the perceived barrier that policy adherence would be limited by capacity for policy knowledge dissemination. Respondents noted that trainings and education about the policy among both ED staff and LEOs would be necessary. Finally, respondents communicated that *leadership buy-in* would be required for effective adoption and enforcement of a policy within both hospitals/healthcare institutions and LEO organizations.

Very few facilitators were identified. The facilitators that were endorsed referred to specific categories of personnel: 1) ED staff (physicians, nurses and other staff); 2) hospital administration; 3) hospital security; 4) LEOs; and 5) social workers. Responses to this question consisted of predictions by respondents on which groups of individuals would be most helpful in implementing a new policy. Interestingly, unlike the multidimensional barriers described in the previous section, respondents did not list non-personnel facilitators to policy adoption (Table 2).

## DISCUSSION

To our knowledge, this is the first study in which a national sample of emergency physicians identified their observations and perceptions on the presence of law enforcement in the ED. The majority of respondents reported that in their experience, there was a regular and frequent (daily or weekly) presence of LEOs in the ED. Most reported that law enforcement presence was helpful to clinicians in the ED as opposed to only 38% who felt it was helpful to patients. The majority of EPs also felt that information-gathering by LEOs was appropriate in the ED setting, especially after completion of a patient’s initial work-up. The meaning behind this difference is beyond the purview of this study but may be influenced by the demographic and experiential context of the cohort that completed the survey. Respondents reported primarily non-Hispanic White and male identities and, thus, the perceptions of LEOs’ activities in the ED may be bounded by their racialized and gendered experiences with LEOs in day-to-day life. Our sample of surveyed EPs was more homogenous than the racial/ethnic composition of practicing EPs and the general US physician workforce, who are estimated to be 69%–73% and 56.2% White, respectively.^8^–^10^ Our study results should be considered within the context of known racial/ethnic discordance between mostly White EPs and the more racially diverse ED patients they serve.

While EPs in our sample endorsed that they had at least some authority to direct LEO access to patients, only 13% were aware of extant policy through which to guide their decisions. The most common factor EPs cited as determinant of authorizing access to patients was the patient’s potential as a threat to public safety. In 2022, ACEP conducted a survey to enumerate the extent of violence exposure that EPs face in the ED. Survey results indicated that EPs have an increased perception of risk of violence posed by patients but do not describe trainings or standardized education that would help them judge and report this risk, highlighting the need for explicit guidance for how and when to engage LEOs.^11^

The facilitators and barriers cited in our results in relation to theoretical policy implementation identify important considerations for building clarity and communication in this area. All respondents listed multiple personnel in the ED who could serve as potential facilitators to the enforcement of policy, for example, triage nurses or physicians. On the other hand, barriers were cited across multiple domains including enforcement, leadership, education, operational challenges, and potential consequences. Doubtless, effective policy in this area must be multidisciplinary and collaborative to appropriately incorporate the interests of patient, clinicians, and law enforcement.

The social context of policing and healthcare in the communities frequently served by the ED is another consideration in policy development, even if implemented in a way that overcomes common barriers. Survey respondents in our study endorsed that LEOs in EDs have a positive influence on the community’s trust in the healthcare institution. This perception prompts the need for additional exploration. Emergency physicians generally have limited information through which to gauge how the communities that use the ED perceive law enforcement presence concurrent in emergency care, other than anecdotal reports. While we could not evaluate the interpretation of how law enforcement presence moderates patient and community trust, it is critical to understand the social meaning of intersections between the healthcare and law enforcement sectors and the communities that both serve.

The way different communities regard healthcare institutions and law enforcement agencies is highly dependent on collective and individual, as well as historical and contemporary experiences. Racialized assumptions that Black Americans are prone to criminality, for example, have been shown to pervade and impact healthcare encounters.^12^,^13^ Assumptions about a patient’s presumed criminality or presumed non-culpability, in the circumstances leading to an ED visit, may influence clinicians’ decisions that guide LEOs’ access to patients. Law enforcement presence at the bedside, in turn, may serve to reinforce discriminatory assumptions and to further erode clinicians’ trust in patients, and vice versa.

The presence of LEOs in the clinical space, whether warranted or not in the context of public safety and criminal legal proceedings, has potential harms. Therefore, EPs should have a working knowledge of relevant ethical considerations. The first area of ethical consideration requires acknowledgment of the consequences of an overlap between racialized inequities in ED utilization and racialized biases that potentiate negative experiences with law enforcement. Due to structural barriers in access to healthcare writ large, Black and Hispanic patient populations have higher rates of ED utilization than their White counterparts; these same groups are most likely to be impacted by racialized over-policing and violence when interacting with law enforcement and the criminal legal system.^14^–^17^ Studies have found that individuals who have contact with the criminal legal system (being stopped by police, arrested, convicted or incarcerated) are less likely to obtain medical care they thought they needed when compared to those who have never been stopped, arrested, convicted, or incarcerated.^18^ Emergency physicians should be familiar with these complex and interdependent realities and the ways in which the presence of law enforcement in EDs is conditioned to, whether directly or indirectly, disproportionately affect Black and Hispanic patients and staff.

A second ethical consideration of importance that emerged in the interpretation of the survey results was that of “dual loyalty,” which refers to the simultaneous obligations, express or implied, to a patient and to a third party (typically the state). This concept is highly relevant considering the ethical ambiguities presented by unregulated LEO presence in the ED.^19^ The International Dual Loyalty Working Group has issued a set of guiding principles. These include the recommendations that health professionals be able to identify situations where dual-loyalty conflicts threaten human and civil rights, and that health professionals protect patient medical confidentiality from state actors whenever possible. Educational and operational leaders in emergency medicine may consider incorporating these guidelines into their development of training curriculum and institutional policies that dictate the scope of LEO activities in the ED.^20^

Survey respondents looked to LEOs as a source of safety for staff, likely due to concerns of workplace violence (WPV) experienced by ED staff. Workplace violence—defined as “incidents where staff are abused, threatened or assaulted in circumstances related to their work, including commuting to and from work, involving an explicit or implicit challenge to their safety, well-being or health”—is a global problem, and the ED has repeatedly been demonstrated to be a high-risk clinical space.^21^ Workplace violence has been associated with numerous negative impacts on the physical and emotional health of healthcare workers and is detrimental to the retention of healthcare workers and the delivery of quality medical care.^22^,^23^ A systematic review on interventions for WPV prevention in the ED reviewed 15 studies exploring behavioral, organizational, and environmental interventions; none of the interventions involved the addition of law enforcement staff.^24^ Instead, recommendations center on preventative measures, such as ensuring adequate staffing and effective triage, improving patient-clinician communication, de-escalation trainings, enforcement of existing policies, and legislation regarding the reporting and filing of charges when appropriate.^25^–^28^ Undoubtedly, ensuring staff safety must be a priority for individual hospitals and for the healthcare workforce at large. However, EPs should be aware that the current body of evidence does suggest that LEO presence prevents WPV. Training EPs on the dual loyalty principle, as well as on the legal, constitutional, and human rights of their patients may allow them to view the presence of LEOs in EDs as an issue distinct from that of staff safety.

As legal scholar Song describes in her recent law review, patients seeking emergency care do not have the same freedoms as individuals on the street to walk away from an encounter due to their medical needs.^5^ Song’s legal and ethical concerns are echoed by clinicians in a recent qualitative study by Harada et al on the understanding of EPs about LEO activity in the ED.^3^ While EPs in this study reported that LEOs could provide helpful information about patients involved in traumatic events, they also reported several ways in which they felt that LEOs interrupted treatment, caused breaches in patient confidentiality, and diminished patient trust in healthcare clinicians and institutions.

Further studies that measure patient perceptions and patient-centered outcomes related to law enforcement presence are important. In a qualitative study by Liebschutz et al, the authors interviewed Black male victims of stabbings and shootings and found institutional mistrust among participants as a result of interactions with police during their medical care.^29^ Participants described suspicion of both police and healthcare. Participants perceived healthcare personnel as allowing police interrogation, which made some feel as if they were being treated as the perpetrator rather than a victim. In a study by Jacoby et al, injured Black patients conveyed mixed feelings about the presence of law enforcement in the ED.^30^ These patients valued police officers’ provision of security at the scene of an injury, assistance in transport to the hospital, and support and information after injury. On the other hand, patients interpreted police questioning as stressful and, at times, disrespectful and in conflict with attention to their emergent clinical needs.

## LIMITATIONS

Our findings must be considered within the limitations of our study. First, while the EMPRN network is designed to mirror the demographics of the ACEP membership, our survey respondents may not reflect the demographics of practicing EPs across the country. Second, we are limited in asserting the generalizability of our findings to all ACEP members given a response rate of 18.4%. However, because our survey was administered as part of a series of surveys on multiple topics, it is unlikely that this response rate introduces nonresponse error related to the survey topic itself. Third, our survey design relies on self-report, which is vulnerable to recall bias as well as social desirability bias. Lastly, our survey does not include the observations and attitudes of other key stakeholders, including ED nurses, technicians, hospital administration and, most importantly, patients and families.

## CONCLUSION

Despite the study’s limitations, we can conclude that while law enforcement activity in the ED is a frequent occurrence, few emergency physicians are aware of institutional policies or guidelines on these interactions. This has the potential to result in ad-hoc decision-making, during which EPs are likely to prioritize staff safety and public safety. Our findings highlight the conflicting interests EPs face when balancing perceived safety with the privacy and autonomy concerns for their patients. Future studies that explore the impacts on patients, clinicians, and the surrounding community of allowing for law enforcement activities in EDs are warranted.

## Supplementary Information





## Figures and Tables

**Table 1:** Demographic characteristics of respondents.

	N	Percent
Region		
Northeast	27	19%
Southeast	33	23%
Southwest	22	16%
Midwest	34	24%
West	25	18%
Age		
Under 35	1	1%
35–45	39	28%
46–55	43	31%
56–65	40	28%
Over 65	18	13%
Race and ethnicity		
Asian	5	4%
Black or African American	1	1%
Hispanic or Latino	1	1%
Other	14	10%
Two or more races	3	2%
White	113	82%
Gender		
Female	27	19%
Male	114	81%

**Table 2:** Perceived barriers and facilitators to policy implementation.

Barriers to policy adherence	Description	Exemplar quote(s)
Public safety	Interfering with police work could harm public safety	“If a crime has been committed and the police need to interact with a patient to get information for public safety, then this is an emergent issue (just as emergent as the patient’s medical issues being emergent). If there was a policy where police could not interact with patients when a crime has been committed, this can be a danger to others in our area (like if a patient was stabbed or shot, and now a potential murderer needs to be found before they hurt someone else).”
Enforcement concerns	Concerns about how and who would enforce policy and whether police would respect policies from within healthcare organizations	“Police often are quite intimidating and cite the reasons they need to access patients and why rules do not apply to them. Standing up to police often results in lots of headache.” “Police ignore it and staff can’t do anything about it.”
Difficult to standardize	Comments on unique situations, and the nuance of emergency setting, which makes creating an applicable and coherent policy difficult	“Cases vary widely and a policy could not cover every scenario, so would be hard to adhere to.”
Education/communication of policy	Concerns about adequate trainings for clinicians and police and communication of policy between hospital and LEO administration	“Lack of communication to the actual officers so they won’t even know the policy” “Providers not knowing the policy and applying it inconsistently”
Leadership buy-in	Concerns regarding the extent to which hospital leadership and administration and LEO leadership and administration would invest in new policy	“If there is no ED leadership involved in creation of the policy barriers will occur. Hospital regulatory and risk do not fully understand the ED environment, especially an environment that can feel like a war zone at times with the amount of violence and trauma seen.” “Unless mutually agreed to in advance by law enforcement and hospital it can lead to increased tension and conflict at the point of care in the ED.”

Facilitators to policy adherence	Description	Exemplar Quote(s)

Personnel	Categories of ED personnel who would aid in adoption and dissemination of an institutional policy	“Nursing staff very much advocate for enforcing written hospital policy.“ “Triage nurse or hospital security would help enforce.” “ED physician and nursing management, law enforcement representatives” “We would need help at several levels--legal, risk management, law enforcement.”

## References

[1] Glauser J (2001). Rationing and the role of the emergency department as society’s safety net. Acad Emerg Med.

[2] Gordon JA (1999). The hospital emergency department as a social welfare institution. Ann Emerg Med.

[3] Harada MY, Lara-Millan A, Chalwell LE (2021). Policed patients: how the presence of law enforcement in the emergency department impacts medical care. Ann Emerg Med.

[4] Lara-Millán A (2014). Public emergency rrom overcrowding in the era of mass imprisonment. Am Sociol Rev.

[5] Song JS (2021). Policing the emergency Room. Harvard Law Review.

[6] Baker EF, Moskop JC, Geiderman JM (2016). Law enforcement and emergency medicine: an ethical analysis. Ann Emerg Med.

[7] (2018). Physicians ACoE. Law Enforcement Information Gathering in the Emergency Department.

[8] Ehrhardt T, Shepherd A, Kinslow K (2021). Diversity and inclusion among U.S. emergency medicine residency programs and practicing physicians: towards equity in workforce. Am J Emerg Med.

[9] Nelson LS, Keim SM, Baren JM (2018). American Board of Emergency Medicine Report on Residency and Fellowship Training Information (2017–2018). Ann Emerg Med.

[10] AAMC (2019). Diversity in Medicine: Facts and Figures 2019.

[11] (2022). ACEP Emergency Department Violence Poll Results.

[12] Muhammad KG (2010). Writing Crime into Race: Racial Criminalization and the Dawn of Jim Crow. The Condemnation of Blackness: Race, Crime, and the Making of Modern Urban America (35–87).

[13] Aronowitz SV, McDonald CC, Stevens RC (2020). Mixed studies review of factors influencing receipt of pain treatment by injured Black patients. J Adv Nurs.

[14] Parast L, Mathews M, Martino S (2022). Racial/ethnic differences in emergency department utilization and experience. J Gen Intern Med.

[15] Gelman A, Fagan J, Kiss A (2007). An analysis of the New York City Police Department’s “stop-and-frisk” policy in the context of claims of racial bias. J Am Stat Assoc.

[16] Agarwal P, Bias TK, Madhavan S (2016). Factors associated with emergency department visits: a multistate analysis of adult fee-for-service medicaid beneficiaries. Health Serv Res Manag Epidemiol.

[17] Snowden LR, Catalano R, Shumway M (2009). Disproportionate use of psychiatric emergency services by African Americans. Psychiatr Serv.

[18] Brayne S (2014). Surveillance and system avoidance. Am Sociol Rev.

[19] Atkinson HG (2019). Preparing physicians to contend with the problem of dual loyalty. J Hum Rights.

[20] Allhoff F (2008). Dual loyalty and human rights in health professional practice: proposed guidelines and institutional mechanisms. Physicians at War: The Dual-Loyalties Challenge (15–38).

[21] Devi S (2020). COVID-19 exacerbates violence against health workers. Lancet.

[22] Watson A, Jafari M, Seifi A (2020). The persistent pandemic of violence against health care workers. Am J Manag Care.

[23] Vento S, Cainelli F, Vallone A (2020). Violence against healthcare workers: a worldwide phenomenon with serious consequences. Front Public Health.

[24] Wirth T, Peters C, Nienhaus A (2021). Interventions for workplace violence prevention in emergency departments: a systematic review. Int J Enviro Res Public Health.

[25] Nelson R (2014). Tackling violence against health-care workers. Lancet.

[26] Liu J, Gan Y, Jiang H (2019). Prevalence of workplace violence against healthcare workers: a systematic review and meta-analysis. Occup Environ Med.

[27] Phillips JP (2016). Workplace violence against health care workers in the United States. N Engl J Med.

[28] D’Ettorre G, Pellicani V, Mazzotta M (2018). Preventing and managing workplace violence against healthcare workers in emergency departments. Acta Biomed.

[29] Liebschutz J, Schwartz S, Hoyte J (2010). A chasm between injury and care: experiences of Black male victims of violence. J Trauma.

[30] Jacoby SF, Branas CC, Holena DN (2020). Beyond survival: the broader consequences of prehospital transport by police for penetrating trauma. Trauma Surg Acute Care Open.

