# Supplementary material for: Emergency Physician Observations and Attitudes on Law Enforcement Activities in the Emergency Department
Source: West J Emerg Med. 2023 Feb 20;24(2):160–8. doi: 10.5811/westjem.2022.12.57098 (PMC10047729; doi:10.5811/westjem.2022.12.57098)
Supplement: Supplementary file 2 [file wjem-24-160-s002.docx]

**Police in the ED**

1. How often have you observed the police interacting with emergency department (ED) patients?
   1. Never (skip to 5)
   2. About once a year
   3. About once a month
   4. About once a week
   5. Several times a week
   6. Every day
2. What have you observed the police doing in the EDs? (check all that apply)
   1. Providing security (not as part of hospital security staff)
   2. Questioning a patient as a witness to a crime
   3. Questioning a patient as a suspect to a crime
   4. Collecting evidence from patients
   5. Collecting or securing property that patients bring into the ED
   6. Accompanying a patient who is agitated, altered or intoxicated
   7. Accompanying a patient who is under arrest
   8. Accompanying a patient who is incarcerated or jailed
   9. Securing the body of a patient who has died
   10. Responding to a call/report from hospital staff
   11. Bringing in a suspect of a crime for identification by a patient
   12. Other <open>

For the next two questions, please use the following scale:

(Likert scale or slider: 1= Almost never 2= Seldom 3= About half the time 4= Usually 5=Almost always)

1. How often have you observed police presence being helpful to *your clinical work* in the ED?
   1. How have you observed policy presence being helpful to your clinical work in the ED? <open>
2. How often have you observed police presence being harmful to *your clinical work* in the ED?
   1. How have you observed policy presence being harmful to your clinical work in the ED? <open>

1. On a continuum from very harmful to very helpful, *for patients,* do you see police presence during the emergency care of ED patients as… (check one)
   1. Very harmful
   2. Harmful
   3. Somewhat harmful
   4. Sometimes helpful and sometimes harmful]
   5. Somewhat helpful
   6. Helpful
   7. Very helpful
2. How do you think the presence of external police (i.e. those not employed by the hospital) in an ED affects the following things? (Likert scale or slider from negative to positive for all categories 1 =Very negative 2: Somewhat negative 3 Neither negative nor positive 4= Somewhat positive 5=Very positive)
   1. Clinician-patient rapport
   2. Provider’s clinical throughput and quality of care
   3. Individual patient’s trust in the health care institution
   4. Surrounding community’s trust in the health care institution
   5. Institution-police system(s) relationships
3. On a continuum from very harmful to very helpful, *for clinical care providers,* do you see police presence during the emergency care of ED patients as… (check one)
   1. Very harmful
   2. Harmful
   3. Somewhat harmful
   4. Sometimes helpful and sometimes harmful
   5. Somewhat helpful
   6. Helpful
   7. Very helpful
4. On a continuum from very harmful to very helpful, *for public/community safety,* do you see police presence during the emergency care of ED patients as… (check one)
   1. Very harmful
   2. Harmful
   3. Somewhat harmful
   4. Sometimes helpful and sometimes harmful
   5. Somewhat helpful
   6. Helpful
   7. Very helpful
5. In your role as an emergency physician, do you feel that you have any oversight or influence over police access to patients in the emergency department?
   1. Yes
   2. No
   3. Don’t know
6. When deciding to allow or not allow police access to your patients during their clinical care, please indicate the importance of… *<For each multiple choice: 1 highly unimportant, 2 unimportant, 3 neither important or unimportant, 4 important, 5 highly important>*
   1. Severity of patient’s condition
   2. Patient’s ability to provide informed consent to interact with law enforcement officers
   3. Patient’s willingness/preference to interact with law enforcement officers
   4. Patient’s potential as threat to public safety
   5. Patient’s ability to provide information that can increase public safety
   6. Safety of emergency department staff
   7. Safety of police and other law enforcement officers
7. In situations where the primary purpose of police presence is information gathering about a crime or suspected crime (i.e. safety of staff or patients is not a concern), when and where do you think it appropriate for police to interact with ED patients? *Check as many as apply.*
   1. Police should not interact with patients in any patient-care area of the hospital
   2. The trauma bay during resuscitation
   3. Trauma bay after initial workup
   4. General Emergency Department during initial workup
   5. General Emergency Department after initial workup
   6. Inpatient floor or ICU after admission
8. Does your institution or hospital have a policy(ies) or guideline that guide(s) police interactions with ED patients?
   1. No
   2. Yes
      1. Briefly describe the policy <open>
   3. I don’t know
9. If a policy were to be adopted by your hospital or institution, do you foresee any barriers preventing its routine adherence?
   1. No
   2. Yes
      1. Briefly describe <open>
   3. I don’t know
10. If a policy were to be adopted by your hospital or institution, do you foresee any facilitators to its routine adherence?
    1. No
    2. Yes
       1. Briefly describe <open>
    3. I don’t know
